# Relationship between Empowering Leadership and Stress in a French University Hospital: A Cross-Sectional Study Combining the Measurement of Perceived Stress and Salivary Cortisol

**DOI:** 10.1155/2024/8839893

**Published:** 2024-01-31

**Authors:** Baptiste Cougot, Nicolas Gillet, Leïla Moret, Jules Gauvin, Pascal Caillet, Ghozlane Fleury-Bahi, Johan Lesot, Florian Ollierou, Anne Armant, Arthur Peltier, Evelyne Fouquereau, Isaac Getz, Kalyane Bach-Ngohou, Dominique Tripodi

**Affiliations:** ^1^Nantes University, Nantes University Hospital, Department of Occupational Medicine and Environmental Health, Nantes F-44000, France; ^2^Nantes University, Nantes University Hospital, Department of Public Health, Nantes F-44000, France; ^3^EE1901 QualiPsy, Department of Psychology, Tours University, Tours F-37000, France; ^4^IGEIA, Audit Intervention Formation Recherche en Santé, Nantes F-44000, France; ^5^Institut Universitaire de France (IUF), Paris F-75000, France; ^6^Nantes University, Tours University, Nantes and Tours University Hospitals, INSERM, Methods in Patients-Centered Outcomes and Health Research, SPHERE, Nantes F-44000, France; ^7^EA4638 LPPL, Department of Psychology, Nantes University, Nantes F-44000, France; ^8^SSTRN “Nantes Region Occupational Health Department”, Nantes F-44000, France; ^9^ESCP Business School, Paris F-75011, France; ^10^Nantes University, Nantes University Hospital, Department of Biochemistry, Nantes F-44000, France; ^11^Nantes University, UMR 1235 INSERM TENS “The Enteric Nervous System in Gut and Brain Disorders”, Nantes F-44000, France

## Abstract

**Aim:**

We investigated the impact of empowering leadership on both perceived stress and salivary cortisol, a commonly utilized biological indicator for stress assessment.

**Background:**

Empowering leadership is gaining increasing interest in companies. However, the impact of empowering leadership on stress is still insufficiently explored, with conflicting findings within the literature on this topic. While certain studies indicate that empowering leadership reduces perceived stress, other studies have suggested that empowering leadership could be stressful.

**Methods:**

We conducted a cross-sectional study using a questionnaire among a sample of 397 participants working in a French hospital. Participants' salivary cortisol was assessed. All analyses exploring the relationships between empowering leadership, perceived stress, and salivary cortisol were performed using multiple imputation methods.

**Results:**

Empowering leadership could simultaneously increase and decrease perceived stress. Specifically, although the empowering leadership global factor showed a negative correlation with perceived stress, some specific empowering leadership behaviors were positively associated with perceived stress. However, salivary cortisol was positively related to perceived stress and strictly negatively related to empowering leadership. Furthermore, salivary cortisol could be explained by a significant interaction effect between perceived stress and empowering leadership, indicating that empowering leadership enables employees to cope with perceived stress.

**Conclusions:**

Although empowering leadership was an ambiguous antecedent of perceived stress, our findings suggested that empowering leadership was a protective factor against increased salivary cortisol. These results suggest that empowering leadership behaviors could prevent biological stress. *Implications for Nursing Management*. While empowering leadership showed a protective effect on salivary cortisol, it is essential for managers to adopt the full set of empowering leadership practices to guarantee protective effects on perceived stress. This trial is registered with NCT04010773.

## 1. Introduction

Managerial practices aiming to foster employees' autonomy, such as empowering leadership, are gaining interest in companies [1, 2]. Unlike traditional leadership behaviors that consist of motivating employees through rewards (e.g., promise of bonuses) or threats (e.g., threat of blame or sanction; [3]), empowering leadership aims to motivate employees by increasing their power over their professional role and settings [4]. Two main categories of behaviors can be distinguished within empowering leadership: behaviors of power sharing with the employees and behaviors of support of the employees' autonomy [4]. Under these managerial conditions, the employees can not only exercise autonomous control over their thoughts and behaviors (i.e., self-leadership) and thus make concrete experiences of self-determination and self-efficacy but also impact and meaningful experiences at work (i.e., psychological empowerment [5]). These psychological experiences are intrinsically satisfying [6], and they, in turn, promote the identification of employees with their work and their increased occupational commitment [7] and improve the company's performance [8].

While the benefit of this type of leadership for the efficiency of companies is well established [8], its impacts on health remain understudied, with conflicting findings within the literature on this topic [9]. On the one hand, empowering leadership was found to reduce stress by fostering resource development and thus enabling the employees to meet their work requirements [10, 11]. However, these effects were low [10, 11]. On the other hand, in contrasting investigations, empowering leadership was suspected to promote occupational stress [12]. More specifically, they have suggested that the autonomy resulting from empowering leadership may represent a cognitive demand that induces stress for employees [12, 13]. Hence, additional studies designed to clarify the impact of empowering leadership on stress are needed. Beyond the scientific interest, this would enable us to make recommendations for the implementation of empowering leadership, with the aim of preventing possible negative effects in terms of stress. By conducting this research, our aim is to further understand how empowering leadership influences stress. More specifically, by developing two original perspectives, we aim to clarify the valence of the association between empowering leadership and stress and to improve our comprehension of the mechanisms underlying this link.

First, we examined the effects of empowering leadership on perceived stress and on salivary cortisol, considered as a biological indicator of stress [14]. As a steroid hormone, cortisol is secreted, under the regulation of the hypothalamic-pituitary-adrenal axis, by the adrenal glands. It helps to regulate energy and cellular metabolism all along the day [15]. Cortisol secretion exhibits cyclic biological variations, increasing in the second part of the night leading to a peak following awakening and a decrease during the day, with the lowest level at night [16, 17]. Upon exposure to a stressful situation, the activation of the hypothalamic-pituitary-adrenal axis intensifies, triggering an increase in secretion. This response aims to prepare the organism to effectively cope with stress [14]. The cortisol level, therefore, appears as a relevant marker of the activation of the biological mechanisms of the stress response [14, 16]. Salivary cortisol level is the most widely used parameter because it measures the free (and active) level of cortisol and because of the simplicity and the noninvasive side of its collection [17, 18]. Although perceived stress triggers the activation of these biological mechanisms [19], it does not capture the biological mechanisms of stress [14, 16]. Accordingly, completing perceived stress measurement with the assessment of salivary cortisol could provide stronger evidence of the effect of empowering leadership on stress. Two alternative models for assessing the association between empowering leadership and salivary cortisol were tested. First, given the precursor role of perceived stress in the activation of biological mechanisms [19], the mediating effect of perceived stress on the relationship between empowering leadership and salivary cortisol was studied.

**Hypothesis 1 .**

The effect of empowering leadership on cortisol will be mediated by perceived stress.

The moderating effect of empowering leadership on the relationship between perceived stress and salivary cortisol was then investigated. Indeed, empowering leadership promotes employees' behavioral engagement in the transformation of their environment in order to develop their resources and their ability to cope with job demands [20]. However, the literature indicates that the effect of perceived stress on health can be mitigated depending on the efficacy of the individuals' coping strategies [21, 22]. Consequently, it may be assumed that empowering leadership will decrease salivary cortisol by allowing employees to effectively cope with perceived stress.

**Hypothesis 2 .**

Empowering leadership will moderate the effect of perceived stress on cortisol, with a weaker effect of perceived stress on cortisol when empowering leadership increases.

In addition, we attempted to distinguish the overall effect of empowering leadership from that of each of its specific component behaviors, using bifactor models [23]. Bifactor analyses consist of specifying both a global factor defined by the entire set of items and the specific factors [23]. Once the global factor has been estimated (i.e., when the globality has been taken into account), the specific factors are estimated using the residual information [23]. As mentioned earlier, prior research has indicated that empowering leadership power-sharing behaviors could be a stressful demand [12]. Conversely, empowering leadership-related supportive behaviors could be an occupational resource, since they would allow employees to deal with the autonomy-related demands [4]. Briefly, empowering leadership might have contrasting effects contingent on the empowering leadership behaviors considered. Such a method could allow highlighting in an original way the possible contrasting impacts of empowering leadership on stress that has been suggested [12]. Considering the lack of previous studies relying on bifactor models to reflect empowering leadership and its effect on stress, we consider the effect of empowering leadership-specific factors on perceived stress and cortisol as an open research question. However, assuming that the bifactor model will be validated, and given the generally positive effects associated with empowering leadership, we expect the empowering leadership global factor to be negatively associated with perceived stress and cortisol.

**Hypothesis 3 .**

The empowering leadership global factor will be negatively associated with perceived stress and cortisol.

## 2. Methods

### 2.1. Study Participants, Procedure, and Cortisol Level Measurement

Between January and May 2018, we conducted a cross-sectional study involving employees working in a French university hospital. Data were collected during the compulsory medical examination within the occupational health department. The participants were included by the physician, provided that they did not work at night and were not pregnant, after having given their written informed consent to participate. After completing the questionnaires, instructions for saliva self-sampling using a salivette (Sarstedt, Marnay, France) were given by a nurse to all participants. They were asked to (a) collect the sample between two working days, to ensure that the salivary cortisol level could reflect both recent occupational exposure and occupational exposure anticipation [24]; (b) avoid collecting the sample after an intense stressful event, to ensure that the salivary cortisol level reflected chronic occupational exposure and not an isolated event [24]; (c) in participants treated with corticosteroids, not to take treatment within 24 hours prior to sample collection [17, 18]; and finally (d) collect the sample in the morning, after awakening, and without toothbrushing, while fasting and without tobacco [17, 18]. Sampling was scheduled after awakening to accommodate the circadian rhythm of cortisol [16, 25].

Salivary cortisol levels were analysed by the physician of the occupational health department for medical control. Among all the participants with an elevated salivary cortisol level, none was diagnosed with Cushing's syndrome [26].

This study was ancillary to the Chrysalide research project [27], so there was no sample size determined specifically for this study. The sample collected (*n* = 397) was deemed sufficient to conduct structural equation modelling for the measurement model [28].

### 2.2. Salivary Cortisol Analyses

Free salivary cortisol level was assessed through a previously described “liquid chromatography-tandem mass spectrometry” (LC-MSMS) in-house method [29]. This method was chosen because of the specificity of its measurement [18]. Briefly, free salivary cortisol was determined from 500 *μ*L of the salivary sample, using a liquid-liquid extraction (LLE) with dichloromethane. High-performance liquid chromatography (HPLC) systems were from Agilent Technology (Agilent Technologies, Les Ulis, France) coupled with 3200 Qtrap spectrometers (Sciex, Les Ulis, France). The quantification transition used was 363 > 121. The deuterium internal standard contained cortisol-d4. Cortisol standards for establishing calibration curves were diluted in methanol.

### 2.3. Psychometric Measures

Empowering leadership was measured using the Leader Empowering Behaviour Questionnaire (LEBQ; Supplementary Table S1) created by Konczak et al. [30]. The questionnaire consists of 17 items helping to measure six behaviors: accountability, delegation of authority, information sharing, self-directed decision-making, coaching for innovative performance, and finally skills development. The level of agreement with each statement was indicated on a seven-point scale, ranging from “strongly disagree” to “strongly agree.” The LEBQ has been found to exhibit satisfactory criterion validity and reliability [30, 31].

Perceived stress was measured using the Perceived Stress Scale in its four-item version [32]. Participants were asked how often they had been faced with each situation during the last four weeks, on a five-point scale ranging from (1) “never” to (5) “often.” The four-item version of the PSS has been previously found to exhibit satisfactory criterion validity and reliability [33].

### 2.4. Ethical Approval Statement

This work belongs to the Chrysalide research project [27]. The Nantes University Hospital Ethics Committee (i.e., GNEDS, Groupe Nantais d'Éthique dans le Domaine de la Santé) approved the research protocol (reference number GNEDS02122018). The entire methodology was conducted in line with the Declaration of Helsinki guidelines. The protocol is registered online under the reference number NCT04010773 (ClinicalTrials.gov).

### 2.5. Statistics

#### 2.5.1. Test of the Bifactor Configuration of Empowering Leadership

We first tested the bifactor structure of empowering leadership using Mplus software, version 8.3. The models were assessed with the maximum likelihood of robust standard errors (MLR). The analysis strategy recommended by Morin et al. [23] was used. More precisely, four competing empowering leadership measurement models were estimated (Supplementary Figure S1 online): (1) the original six correlated factors model is proposed by Konczak et al. [30] using confirmatory factor analysis (CFA), in which the factors are strictly defined by their corresponding items; (2) the same six correlated factors configuration using exploratory structural equation model (ESEM), in which the items can contribute to multiple dimensions simultaneously as cross-loadings are considered [23]; (3) a configuration using a bifactor CFA model (BCFA) with one global factor defined by the entire set of empowering leadership items and six specific factors defined by their respective items [23] (Factors were specified as independent (i.e., orthogonal) without cross-loadings, considering that the global factor accounts for the covariance between the dimensions [23]); and (4) the same configuration using a bifactor-ESEM model (BESEM), in which the items are allowed to contribute to the other specific factors, as well as to the global factor and their dedicated specific factor. The best factor configuration for measuring empowering leadership was then used to assess the complete measurement model integrating empowering leadership and perceived stress (defined based on a CFA model).

We then compared the models with each other, assessing the parameters and fit indices estimated within the models. The model fit was evaluated by utilizing the root mean square error of approximation (RMSEA), comparative fit index (CFI), and Tucker–Lewis index (TLI). A CFI (or a TLI) equal to or exceeding 0.90 indicates an acceptable fit, while a value of 0.95 or higher indicates an excellent fit to the data [34]. Similarly, a RMSEA less than 0.08 is considered acceptable, and a value below 0.06 indicates an excellent fit [34]. The comparison begins with ESEM and CFA models. The ESEM model could be selected based on three criteria: (a) exhibiting the best-fit indices, (b) a decrease in interfactor correlations when accounting for cross-loadings, and (c) a correct definition of factors [34]. Second, a comparison was made between the chosen ESEM or CFA model and its respective bifactor model. One could select the bifactor model based on three criteria: (a) a correct definition of the global factor by the entire set of items, (b) a correct definition of the specific factors, and (c) the best-fit indices [23]. The composite reliability coefficient omega (*ω*) was also calculated for each factor.

#### 2.5.2. Multiple Imputation Strategy

Of the 397 study participants, 110 did not provide their saliva sample (i.e., 28%). To take into account missing data, all the analyses exploring the relationships between empowering leadership, perceived stress, and salivary cortisol were performed using the multiple imputation method [35] using the factor scores (in their standardized form: mean = 0 and standard deviation = 1) extracted from the complete measurement model integrating empowering leadership and perceived stress. The 4.1.0 version of the R software was used to perform analyses with the “mice” package [35]. More precisely, 20 datasets were created by imputing the salivary cortisol level each time using a stochastic regression [35]. Then, the graphical appearance of the 20 imputed distributions of the salivary cortisol level was compared with that of the observed distribution of the salivary cortisol level to verify their similarity and the absence of aberrant imputations (e.g., negative values [35]). Finally, combined regression analyses were performed on the imputed 20 datasets according to the strategy described below.

#### 2.5.3. Relationship between Empowering Leadership, Perceived Stress, and Salivary Cortisol Level

We assessed a series of models to investigate the effect of empowering leadership (a) on perceived stress and (b) on salivary cortisol, (c) the mediating effect of perceived stress on the relationship between empowering leadership and salivary cortisol, and (d) the moderating effect of empowering leadership on the relationship between perceived stress and salivary cortisol. First, two explanatory perceived stress models were assessed: Mps0, with the control variables only, and Mps1, with the addition of the six specific factors and the global factor for empowering leadership as explanatory variables. Mps1 allowed investigation of the effect of empowering leadership on perceived stress. The comparison of the *R*^2^ of Mps1 and Mps0 showed the share of perceived stress variance explained by empowering leadership, independently of the control variables. Then, we assessed a series of explanatory models for salivary cortisol: Mc0 with the control variables only, Mc1 with perceived stress, Mc2 with the specific factors and the global factor for empowering leadership, Mc3 with perceived stress, the six specific factors and the global factor for empowering leadership, and Mc4, using the same specifications as for Mc3, with the interaction between perceived stress and the specific factors, as well as the global factor for empowering leadership. Mc1 showed the effect of perceived stress on salivary cortisol. Mc2 showed the effect of empowering leadership on salivary cortisol. Mc3 showed the relative contribution of perceived stress and empowering leadership in explaining salivary cortisol. Finally, Mc4 showed the interaction between empowering leadership and perceived stress to explain salivary cortisol. The comparison of the *R*^2^ of Mc1 and Mc2 with the *R*^2^ of Mc0 showed the share of salivary cortisol variance explained, respectively, by perceived stress and empowering leadership, independently of the control variables.

All models assessed with multiple imputations were adjusted for the controlled variables (i.e., gender, age, occupation, working time, and length of service in the organization). Fourteen participants reported being treated with corticosteroids (3 oral, 6 dermal, and 5 nasal). Therefore, the explanatory models for salivary cortisol were also adjusted for the intake of corticosteroids. A significance threshold of 5% for the risk of error *α* was applied when testing the hypotheses.

Considering recent developments in statistics, the mediating effect of perceived stress was directly tested using the bootstrapping method [36] combined with multiple imputation analyses [37]. These analyses were performed using bememLavaan package with R [38]. The method involves drawing B bootstrap samples from the original data and then generating K multiple imputations nested within each of the B bootstrap samples. The mediation effect is estimated in each of the *K* *∗* *B* subsamples, giving the distribution of the mediation effect and allowing 95% confidence intervals to be constructed (for details, refer to [37]). According to the recommendations, we performed 1000 bootstrap samples with 20 multiple imputations each [35, 37].

## 3. Results

### 3.1. Participants

The sociodemographic variables and salivary cortisol, perceived stress, and empowering leadership before modelling are described in Supplementary Table S2 online. A sample of 397 participants was included. The mean age was 40.37 ± 10.57 years (range: 19–69 years), and the mean service length in the organization was 13.71 ± 9.81 years. About 80% of participants were women. The most represented occupations were nurses, nurse assistants, and physicians, accounting for 36.3%, 22.2%, and 21.9% of the sample, respectively. Most participants worked full time (71.5%). The mean total empowering leadership score was 4.61 ± 0.95 (range: 1–7), indicating that overall, the participants perceived that their manager was engaged in empowering leadership behaviors. The mean perceived stress score was 6.16 ± 3.25 (range: 0–16), close to that observed in the broader French workforce [39]. The mean salivary cortisol level was 2.84 ± 1.60 ng/mL (range: 0.24–13.50 ng/mL).

### 3.2. Fit of the Measurement Models for Empowering Leadership

Table 1 displays the fit indices of the measurement models. The residual item variance (*δ*), the standardized item factor loadings (*λ*), and the dimension reliability are shown in Supplementary Table S3 online. Supplementary Table S4, available online, presents the correlations between the factors within both the ESEM and CFA models. There was an overall satisfactory fitting for the four empowering leadership measurement models, except for the CFA configuration, with a TLI of 0.889. The best fit to the data for all the indices was obtained with the BESEM model.

### 3.3. ESEM versus CFA Models

The ESEM model exhibited a better fit compared to the CFA one. Both in the ESEM and CFA models, the dimensions for empowering leadership were well-defined by their devoted items (*λ* = 0.289–0.949 and *λ* = 0.360–0.902, respectively). Regarding the ESEM configuration, many cross-loadings could be observed and were found to remain lower than the loading coefficients observed for the devoted items (*λ* = 0.003–0.287). Finally, relative to the CFA model, the ESEM model exhibited a decrease in the interfactor correlations. In sum, the ESEM configuration could be considered the best model.

### 3.4. ESEM versus BESEM Models

The BESEM model was overall well-defined. Except for item 6 relating to accountability (*λ* = 0.053), all empowering leadership items showed high and significant loading coefficients for the global factor (*λ* = 0.357–0.735). The specific factors were found to be correctly defined by their devoted items (*λ* = 0.073–0.708). However, item 7 showed a nonsignificant and very low loading coefficient in defining its specific factor (i.e., self-directed decision-making). Nonetheless, the other dedicated items correctly defined the self-directed decision-making dimension (*λ* = 0.592–0.595). The other specific factors for empowering leadership were also well-defined. In summary, the BESEM model exhibited the best fit, with the global and specific factors being correctly defined. Therefore, the BESEM model was retained as the best model for measuring empowering leadership in further analyses.

### 3.5. Perceived Stress and Empowering Leadership Measurement Model

The final measurement model, combining the BESEM model for measuring empowering leadership and perceived stress, was well-defined (Table 2) and showed an excellent fit for all the indices (Table 1).

### 3.6. SC Level Imputation

The distributions of the salivary cortisol level in the 20 imputed databases, as well as the observed distribution of the salivary cortisol level, are shown in Supplementary Figure S2 online. The appearance of the imputed distributions was similar to that of the observed distribution, without outliers.

### 3.7. Empowering Leadership and Perceived Stress

Table 3 presents the Msp0 and Msp1 models for perceived stress. As expected, the global factor for empowering leadership is negatively correlated with perceived stress, thereby confirming Hypothesis 3. However, the specific factors for coaching for innovative performance, information sharing, and self-directed decision-making were surprisingly positively associated with perceived stress. Empowering leadership accounted for 13.3% of perceived stress. Further analyses revealed a percentage of perceived stress equivalents for the global factor and the specific factors for empowering leadership grouped together, ranging between 5.7% and 7.1% (Supplementary Table S5).

### 3.8. Empowering Leadership, Perceived Stress, and Salivary Cortisol

The models for salivary cortisol are presented in Table 4. A positive correlation was observed between salivary cortisol and perceived stress. Conversely, the global factor for empowering leadership is negatively correlated with salivary cortisol, thereby confirming Hypothesis 3. Unexpectedly, there was also a negative correlation between the specific factor for accountability and salivary cortisol. The comparison of Mc2 with Mc0 revealed that empowering leadership significantly explained 9.8% of the change in salivary cortisol, an explained variance level significantly higher by 8.3% compared to that of perceived stress (comparison of the *R*^2^ of the Mc1 and Mc2 models). Therefore, at this stage, empowering leadership could be considered a stronger predictor of salivary cortisol level after awakening than perceived stress.

Bootstrapping analyses combined with multiple imputations for the test of the mediating effect of perceived stress were not significant (Supplementary Table S6). Therefore, we could not conclude that perceived stress mediated the relationship between empowering leadership and salivary cortisol. Consequently, Hypothesis 1 is rejected.

**Figure 1:**
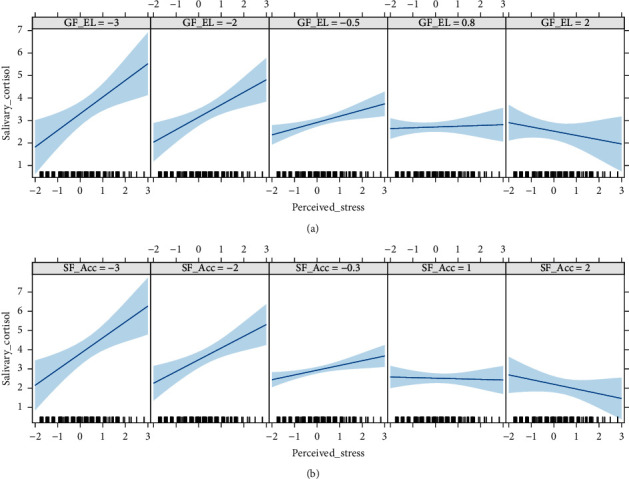
Moderating effect of empowering leadership in the analysis of the relationship between perceived stress and salivary cortisol. (a) Moderating effect of the global factor for empowering leadership. (b) Moderating effect of the specific factor for accountability. Note. GF EL = empowering leadership global factor; SF Acc = accountability specific factor.

However, supporting Hypothesis 2, salivary cortisol could be explained by a significant interaction effect between perceived stress and the specific factor for accountability and the global factor for empowering leadership. The two interaction effects are presented graphically in Figure 1. It revealed that the positive relationship between perceived stress and salivary cortisol decreased as the specific factor for accountability and the global factor for empowering leadership increased, to the extent that when the levels of the specific factor for accountability and global factor for empowering leadership were the highest, perceived stress was no longer related to high salivary cortisol levels.

## 4. Discussion

### 4.1. Bifactor Model for Measuring Empowering Leadership

These findings unequivocally highlighted the superiority of the bifactor model in capturing empowering leadership, thus supporting the simultaneous manifestation of global and specific phenomena within empowering leadership. On the one hand, the identification of the specific factors supported the specific phenomenon, which involves the independent implementation of the behaviors of coaching for innovative performance, skills development, information sharing, self-directed decision-making, accountability, and delegation of authority. On the other hand, the identification of the global factor supported the global phenomenon, which involves the implementation of a set of empowering leadership behaviors. By extension, these findings suggest that managers can selectively exhibit each of the specific empowering leadership behaviors and adopt all empowering leadership behaviors.

Finding a global factor aligns with existing literature. In fact, the most recent scales for measuring empowering leadership propose the combination of various behaviors into overarching factors (e.g., the two-dimensional scale proposed by Amundsen and Martinsen [4]). Nevertheless, the identification of the specific factors along with the global factor raised questions about the limits of the parsimonious operationalization of empowering leadership. Indeed, it could be assumed that they did not allow to take into account the specific nature of certain practices and their outcomes.

### 4.2. Empowering Leadership and Perceived Stress

As expected, the bifactor analysis confirms the contrasting effects of empowering leadership on perceived stress suggested in the literature [12]. We showed that all the empowering leadership behaviors (i.e., the global factor) contributed to decrease perceived stress. This result suggests that empowering leadership functions as an occupational resource that can enhance employees' perceived ability to cope with job demands [40]. This aligns with recent studies on the topic, including those conducted by Kim and Beehr [20] as well as Tripathi and Bharadwaja [11]. However, when the effect of the global factor was taken into account, some specific factors were positively associated with perceived stress. This result confirmed that some empowering leadership behaviors might also pose a stress-inducing demand for employees [40], as proposed by Cheong et al. [12].

First, the positive effect of the specific factor for self-directed decision-making indicated that fostering autonomy and the expectation of autonomy expressed by the manager in the decision-making could be a factor for perceived stress. This finding is aligned with the assumption of Cheong et al. [12], according to which empowering leadership-induced autonomy could be a stressing demand. Furthermore, it is consistent with the mixed effect of autonomy reported in the literature. While it is predominantly viewed as a resource [40], job autonomy is also demanding for employees, as they are required to independently make decisions with regard, for instance, to the work procedure or method [13].

Similarly, the specific factor for coaching for innovative performance was positively related to perceived stress. This could be explained by the fact that encouraging innovation could be demanding [40] for the employees. Indeed, the manager's incentive to propose new ideas implies an effort by the employees to identify problems, to search and encode information, to generate new ideas, and to express them publicly [7]. Therefore, it seems reasonable to consider that the manager's encouragement to innovate in empowering leadership could be demanding for the employees and could, therefore, reduce their resources and generate perceived stress [40].

Finally, the positive association between the specific factor for information sharing and perceived stress indicated that access to information could also be a job demand. In this sense, some studies have shown that the increase in available information may exceed the processing capacities of individuals and lead to a relative experience of information overload and perceived stress [41]. The overload would be observed in particular when the information available is useful [41]. The perceived usefulness would push individuals to consider the information with a potential risk of exceeding their cognitive abilities and experiencing stress [41]. This mechanism could explain the contrasting effect we observed. Once the positive effect of information sharing is taken into account through the effect of the global factor, the sharing of “additional” information could be a factor leading to information overload and thus to perceived stress, characterized by the positive effect of the specific factor for information sharing on perceived stress.

More generally, we could assume that an imbalance mechanism in the implementation of empowering leadership behaviors could also be considered to understand the negative effect of the specific factors [42]. Indeed, it should be noted that the specific factors should be considered as deviations from the global factor for empowering leadership [23]. In other words, we could assume that the global factor reflects the combined implementation of the entire set of empowering leadership behaviors, while the specific factors reflect the isolated implementation of these behaviors. Following this reasoning, the negative effect of the global factor on perceived stress could indicate the protective effect of the combination of the whole set of empowering leadership behaviors, while the positive effect of the specific factors on perceived stress could reflect the demanding effect associated with the isolated implementation of these behaviors. In this sense, the literature widely indicates that the effect of job demands on stress is likely to be moderated by job resources [40]. Using this reasoning, we could assume that the combined implementation of all the empowering leadership behaviors (i.e., the global factor) is likely to moderate the negative effects of information sharing and incentives for autonomy and innovation. Indeed, we can suppose that combining information sharing and incentives for autonomous decision-making and innovation on the one hand, with power sharing, accountability, and skill development on the other hand, could give employees the power and skills they need to cope effectively with the demands of autonomous decision-making, innovation, and information processing and thus reduce perceived stress. Conversely, the selective implementation of these behaviors could induce perceived stress through the mechanisms suggested in the previous paragraphs. Nevertheless, further person-centered research (e.g., latent profile analyses) is needed to confirm this assumption [42].

### 4.3. Empowering Leadership and Salivary Cortisol

Nevertheless, the contrasting effect of empowering leadership seemed limited to the field of perception. Indeed, our results clearly indicated a negative effect of empowering leadership on salivary cortisol. Therefore, our study confirmed the protective effect of empowering leadership against employees' stress. Moreover, with almost 10% of variance explained, empowering leadership was a better predictor of salivary cortisol than perceived stress (1.5%). Thus, the measurement of the employees' resources, and in particular empowering leadership, could be preferred to estimate the impact of the psychosocial environment on their biological health [43, 44].

To understand this effect on salivary cortisol, we tested two alternative models: a perceived stress-mediated empowering leadership model, and a perceived stress-empowering leadership interaction model. Only the interaction model was validated. To put it differently, the protective effect of empowering leadership against elevated salivary cortisol could be better explained by the improved capability of employees to manage perceived stress, rather than by the reduction in perceived stress. In this sense, the transactional model of stress that measures perceived stress at a time *t* could not affect health at a time *t* + 2 if the strategies implemented in the meantime (i.e., at *t* + 1) have allowed regulating the environmental threat and reducing perceived stress [21]. To this end, strategies centered on stress factor regulation generally show a significant efficiency [45]. However, implementing these strategies assumes that the individuals have a sufficient level of resources [43], particularly in terms of control over their environment [22]. Considering the effect of empowering leadership on empowerment and the development of new resources, we could assume that empowering leadership gives employees the resources necessary for their behavioral commitment in the regulation of environmental demands to cope with perceived stress and thus contributes to reduce salivary cortisol.

Besides the effect of the global factor for empowering leadership, we observed that the specific factor for accountability was negatively associated with salivary cortisol. This result is particularly original since the literature suggests on the contrary that accountability could have the effect of a stressful occupational demand [12]. Moreover, the explanatory power of the specific factor for accountability (*b* = −0.405) was significantly higher than that of the global factor for empowering leadership (*b* = −0.246), suggesting that the employees' accountability could be the most protective aspect of empowering leadership against biological stress.

Laboratory studies have shown that the threats of social esteem or identity loss would be a powerful predictor of the activation of the biological response to stress [19]. To address these situations, individuals with high self-esteem tend to lack self-esteem less when faced with a threat to their social identity (e.g., social rejection) and, in turn, to experience a lesser increase in cortisol level [46]. According to us, accountability promoted by the manager could increase self-esteem, by encouraging employees to personally appropriate the results of their work. In this sense, the literature indicates that some forms of support, consisting of indicating to the employees their skills and value for the organization, could have a direct positive effect on self-esteem [47]. This positive effect of accountability on self-esteem could, in turn, reduce the employees' propensity to lack self-esteem, especially when faced with situations likely to threaten their social identity (e.g., observation of their work by others [19]), and thus decrease the activation level of the biological mechanisms of the stress response [46]. Moreover, as a personal resource [40], the self-esteem induced by accountability could also promote the employees' commitment to the regulation of perceived stress [43] and thus reduce the impact of perceived stress on biological stress.

### 4.4. Limitations

Considering that the cultural and organizational contexts can modify the effect of empowering leadership [1], these findings should be replicated in other samples. Furthermore, the cross-sectional design of the study might be biased. An experimental study assessing the effect of different empowering leadership behaviors on several measurements of salivary cortisol upon awakening could provide a stronger level of evidence of the effect of empowering leadership on biological stress [16].

## 5. Conclusions

We can conclude that empowering leadership is an ambiguous predictor of perceived stress, likely to both decrease it but also increase it if specific factors are taken into account. In this regard, these findings underscore the importance of embracing all empowering leadership behaviors to yield positive impacts on stress perception. On the other hand, empowering leadership shows clear protective effect against the increase in salivary cortisol. Although these results need to be confirmed, they suggest that empowering leadership behaviors could prevent biological stress [16].

## 6. Implications for Nursing Management

This study supports the need to implement empowering leadership in organizations and to prevent employee's stress. However, it invites to combine all empowering leadership behaviors to favor protective effects on perceived stress. Specifically, before sharing information and inviting employees to be innovative and autonomous, managers should be willing to recognize responsibility, provide support, and share power. On the flip side, managing through incentives for autonomy and innovation or information sharing, without recognizing employees' responsibility, supporting, and sharing power, might contribute to perceived stress. Therefore, this study calls for future interventions designed to encourage the implementation of the full set of empowering leadership behaviors.

## Figures and Tables

**Table 1:** Results of the fit analysis of the measurement models.

	*χ* ^2^ (df)	CFI	TLI	RMSEA	90% CI
Alternative models of empowering leadership					
CFA	316.244 (104)^*∗*^	0.915	0.889	0.072	[0.063; 0.081]
BCFA	249.763 (102)^*∗*^	0.941	0.922	0.060	[0.051; 0.070]
ESEM	79.874 (49)^*∗*^	0.988	0.966	0.040	[0.023; 0.055]
BESEM	51.744 (38)^*∗*^	0.995	0.980	0.030	[0.000; 0.049]
Measurement model for measuring empowering leadership (BESEM) and perceived stress (CFA)					
	174.937 (101)^*∗*^	0.997	0.952	0.043	[0.032; 0.053]

*Note*. ^*∗*^*p* < 0.01; ESEM = exploratory structural equation modelling; BESEM = bifactor-ESEM; CFA = confirmatory factor analysis; BCFA = bifactor CFA; *χ*^2^ = Khi^2^ test; df = degree of freedom; RMSEA = root mean square error of approximation; TLI = Tucker–Lewis index; CFI = comparative fit index; 90% CI = 90% confidence interval for the RMSEA.

**Table 2:** Standardized parameter estimates (loadings *λ*; residuals *δ*) for measuring empowering leadership (BESEM) and perceived stress (CFA).

Items	BESEM of EL								CFA of PS	
	GF-*λ*	SF-*λ*	SF-*λ*	SF-*λ*	SF-*λ*	SF-*λ*	SF-*λ*	*δ*	*λ*	*δ*
Delegation of authority										
Item 1	**0.698**	**0.555**	*−0.011*	*0.019*	*−0.020*	*0.004*	*0.013*	0.204		
Item 2	**0.696**	**0.626**	*0.028*	*0.034*	*0.024*	*−0.013*	*−0.001*	0.121		
Item 3	**0.673**	**0.224**	0.245	*−0.040*	*0.022*	*−0.061*	*0.134*	0.413		
*ω*		0.728								
Accountability										
Item 4	**0.495**	0.145	**0.614**	*0.108*	*0.087*	*−0.033*	*0.073*	0.331		
Item 5	**0.377**	*0.003*	**0.667**	*0.004*	−0.129	*−0.043*	−0.152	0.372		
Item 6	**0.060**	*−0.057*	**0.437**	0.253	*0.066*	*−0.006*	*−0.088*	0.726		
*ω*			0.674							
Self-directed decision-making										
Item 7	**0.747**	*−0.085*	*−0.063*	**0.063**	*−0.134*	−0.156	*−0.133*	0.367		
Item 8	**0.361**	0.133	0.273	**0.603**	*−0.023*	*−0.062*	*−0.010*	0.409		
Item 9	**0.626**	−0.090	*0.032*	**0.577**	*−0.001*	*−0.001*	*0.012*	0.265		
*ω*				0.597						
Information sharing										
Item 10	**0.721**	*0.050*	*0.020*	*0.017*	**0.419**	0.105	*−0.002*	0.291		
Item 11	**0.689**	*−0.022*	*−0.021*	*−0.051*	**0.580**	*0.071*	*−0.060*	0.178		
*ω*					0.680					
Skills development										
Item 12	**0.505**	−0.124	*−0.030*	0.141	*0.101*	**0.158**	*−0.020*	0.673		
Item 13	**0.653**	*−0.004*	*−0.059*	*−0.032*	*0.030*	**0.659**	*−0.004*	0.134		
Item 14	**0.570**	*0.006*	*−0.017*	−0.105	*0.106*	**0.464**	0.138	*0.418*		
*ω*						0.573				
Coaching for innovative performance										
Item 15	**0.568**	0.083	−0.134	*0.016*	−0.117	*0.068*	**0.414**	0.462		
Item 16	**0.677**	*0.041*	*−0.053*	*0.046*	*0.042*	0.135	**0.303**	0.423		
Item 17	**0.724**	*−0.043*	*−0.101*	−0.122	*0.000*	*−0.048*	**0.230**	0.394		
*ω*	0.940						0.412			
Perceived stress										
Item 1									**0.782**	0.389
Item 2									**0.735**	0.460
Item 3									**0.713**	0.492
Item 4									**0.756**	0.429
*ω*									0.834	

*Note*. PS = perceived stress; EL = empowering leadership; BESEM = bifactor exploratory structural equation modelling; CFA = confirmatory factor analysis; SF = specific factor estimated as part of a bifactor model; GF = global factor estimated as part of the bifactor model; *ω* = omega coefficient of model-based composite reliability; *δ* = item uniqueness; *λ* = factor loading. Target BESEM factor loadings are indicated in bold; nonsignificant parameters (*p* > 0.05) are shown in italic.

**Table 3:** Hierarchical linear model for measuring perceived stress.

	Mps0			Mps1		
	*b*	*s.e.*	*p*	*b*	*s.e.*	*p*
Intercept	−0.01	0.27	0.983	0.14	0.26	0.589
Male versus						
Female	0.02	0.14	0.894	0.03	0.13	0.815
Age	0.01	0.01	0.186	0.00	0.01	0.527
Position N-assistants versus						
HSW	−0.19	0.27	0.478	−0.12	0.26	0.639
Others	−0.45	0.26	0.082	−0.49	0.25	0.050
Nurse managers	−0.47	0.24	0.050	−0.39	0.23	0.086
Head physicians	−0.29	0.43	0.505	−0.11	0.41	0.784
Nurses	−0.17	0.12	0.184	−0.21	0.12	0.087
Physicians	−0.10	0.15	0.538	−0.19	0.15	0.212
Secretaries	0.03	0.21	0.879	0.01	0.20	0.953
Working full time versus						
Part-time	−0.19	0.11	0.079	−0.16	0.10	0.123
Seniority	−0.01	0.01	0.052	−0.01	0.01	0.165
GF for EL				**−0.27**	**0.05**	**0.000**
SF for dele.				−0.03	0.05	0.595
SF for Acc.				0.00	0.05	0.996
SF for self.				**0.17**	**0.05**	**0.002**
SF for info.				**0.13**	**0.05**	**0.017**
SF for skills.				0.04	0.05	0.424
SF for innov.				**0.31**	**0.07**	**0.000**
*R* ^2^			0.039			0.172
Δ*R*^2^						**0.133**

*Note*. Significant parameters are indicated in bold (*p* ≤ 0.05); *b* = nonstandardized regression coefficient; s.e. = standard error of the coefficient; *p*=*p* value; N-assistants = nursing assistants; *R*^2^ = percentage of variance explained; Δ*R*^2^ = difference in variance explained; EL = empowering leadership; Inov. = coaching for innovative performance; Skills. = skills development; Info. = information sharing; Self. = self-directed decision-making; Acc. = accountability; Dele. = delegation of authority; GF = global factor; SF = specific factor; HSW = hospital service workers.

**Table 4:** Hierarchical linear model for assessing the cortisol level (combined analysis of 20 imputed data sets).

	Mc0			Mc1			Mc2			Mc3			Mc4		
	*b*	*s.e.*	*p*	*b*	*s.e.*	*p*	*b*	*s.e.*	*p*	*b*	*s.e.*	*p*	*b*	*s.e.*	*p*
Intercept	**1.89**	**0.67**	**0.006**	**1.88**	**0.67**	**0.006**	**2.22**	**0.64**	**0.001**	**2.20**	**0.64**	**0.001**	**2.13**	**0.64**	**0.001**
Male versus															
Female	0.11	0.31	0.737	0.10	0.31	0.746	0.04	0.31	0.894	0.04	0.30	0.908	0.11	0.30	0.714
Age	−0.02	0.01	0.197	−0.02	0.01	0.148	−0.02	0.01	0.094	−0.02	0.01	0.082	−0.02	0.01	0.066
C-therapy NA versus															
Yes	0.42	0.65	0.515	0.46	0.65	0.476	0.04	0.63	0.949	0.04	0.63	0.944	0.21	0.62	0.735
No	0.60	0.45	0.191	0.61	0.45	0.179	0.31	0.44	0.483	0.30	0.43	0.488	0.35	0.43	0.423
Position N-assistants versus															
HSW	−0.05	0.57	0.937	0.00	0.56	0.995	0.02	0.55	0.977	0.04	0.55	0.945	−0.03	0.55	0.960
Others	0.58	0.53	0.271	0.68	0.53	0.202	0.64	0.52	0.222	0.73	0.53	0.170	0.75	0.52	0.154
Nurse managers	0.51	0.44	0.257	0.60	0.44	0.176	0.80	0.43	0.064	**0.87**	**0.43**	**0.045**	**0.87**	**0.43**	**0.043**
Head physicians	**1.81**	**0.89**	**0.043**	**1.87**	**0.88**	**0.035**	**2.07**	**0.86**	**0.018**	**2.09**	**0.86**	**0.016**	**2.27**	**0.87**	**0.010**
Nurses	−0.01	0.23	0.982	0.03	0.22	0.892	0.23	0.22	0.294	0.27	0.22	0.224	0.22	0.22	0.323
Physicians	**0.81**	**0.32**	**0.013**	**0.83**	**0.32**	**0.011**	**0.93**	**0.32**	**0.005**	**0.97**	**0.32**	**0.003**	**0.98**	**0.32**	**0.003**
Secretaries	0.52	0.39	0.177	0.52	0.38	0.179	**0.77**	**0.39**	**0.049**	**0.76**	**0.38**	**0.048**	0.72	0.38	0.059
Working full time versus															
Part-time	0.30	0.20	0.141	0.34	0.20	0.094	0.28	0.20	0.162	0.30	0.20	0.124	0.31	0.20	0.119
Seniority	**0.04**	**0.01**	**0.001**	**0.05**	**0.01**	**0.001**	**0.05**	**0.01**	**0.001**	**0.05**	**0.01**	**0.000**	**0.05**	**0.01**	**0.000**
GF for EL							**−0.25**	**0.10**	**0.011**	**−0.20**	**0.10**	**0.040**	−0.17	0.10	0.093
SF for dele.							−0.08	0.10	0.442	−0.07	0.10	0.471	−0.10	0.10	0.317
SF for acc.							**−0.40**	**0.12**	**0.001**	**−0.40**	**0.12**	**0.001**	**−0.29**	**0.13**	**0.026**
SF for self.							−0.18	0.11	0.094	−0.21	0.11	0.054	−0.20	0.11	0.081
SF for info.							−0.08	0.11	0.468	−0.10	0.11	0.350	−0.08	0.11	0.447
SF for skills.							0.18	0.10	0.075	0.18	0.10	0.085	0.17	0.10	0.103
SF for innov.							0.02	0.16	0.907	−0.04	0.16	0.824	0.02	0.16	0.925
PS				**0.21**	**0.10**	**0.028**				0.18	0.10	0.079	0.13	0.10	0.185
PS × GF for EL													**−0.18**	**0.09**	**0.039**
PS × SF for dele.													−0.01	0.10	0.952
PS × SF for acc.													**−0.22**	**0.09**	**0.021**
PS × SF for self.													−0.10	0.10	0.355
PS × SF for info.													0.01	0.10	0.945
PS × SF for skills.													0.04	0.12	0.751
PS × SF for innov.													0.01	0.15	0.924
*R* ^2^			0.103			0.118			0.201			0.210			0.251
Δ*R*^2^ with Mc0						**0.015**			**0.098**			**0.107**			**0.148**
Δ*R*^2^ with the previous model						**0.015**			**0.083**			0.009			0.041

*Note*. Significant parameters are indicated in bold (*p* ≤ 0.05); *b* = nonstandardized regression coefficient; s.e. = standard error of the coefficient; *p*=*p* value; C-therapy = corticosteroid therapy; N-assistants = nursing assistants; HSW = hospital service workers; EL = empowering leadership; Inov. = coaching for innovative performance; Skills. = skills development; Info. = information sharing; Self. = self-directed decision-making; Acc. = accountability; Dele. = delegation of authority; SF = specific factor; GF = global factor; PS = perceived stress; *R*^2^ = percentage of variance explained; Δ*R*^2^ = difference in variance explained. All estimated parameters were obtained from the combined analysis of 20 imputed data sets.

## Data Availability

The dataset collected in the study will be available from the corresponding author upon reasonable request three years after the end of the study.

## Supplementary Materials

Supplementary Materials Supplementary Figure S1: alternative models for measuring empowering leadership. Supplementary Figure S2: distributions of the imputed (red) and observed (blue) cortisol levels. Supplementary Table S1: Leader Empowering Behavior Questionnaire (LEBQ). Supplementary Table S2: sample characteristics. Supplementary Table S3: standardized parameter estimates (loadings *λ*; residuals *δ*) for the CFA, BCFA, ESEM, and BESEM models for measuring empowering leadership. Supplementary Table S4: correlations between the latent factors in the CFA and ESEM models. Supplementary Table S5: the hierarchical linear model of perceived stress: distinction between the global factor and the specific factors for empowering leadership. Supplementary Table S6: bootstrapping analyses combined with multiple imputations for the test of the mediating effect of perceived stress.

## References

[1] Cheong M., Yammarino F. J., Dionne S. D., Spain S. M., Tsai C.-Y. (2019). A review of the effectiveness of empowering leadership. *The Leadership Quarterly*.

[2] Getz I. (2009). Liberating leadership: how the initiative-freeing radical organizational form has been successfully adopted. *California Management Review*.

[3] McGregor D. (1960). *The Human Side of enterprise*.

[4] Amundsen S., Martinsen Ø. L. (2014). Empowering leadership: construct clarification, conceptualization, and validation of a new scale. *The Leadership Quarterly*.

[5] Amundsen S., Martinsen Ø. L. (2015). Linking empowering leadership to job satisfaction, work effort, and creativity: the role of self-leadership and psychological empowerment. *Journal of Leadership and Organizational Studies*.

[6] Thomas K. W., Velthouse B. A. (1990). Cognitive elements of empowerment: an “interpretive” model of intrinsic task motivation. *Academy of Management Review*.

[7] Zhang X., Bartol K. M. (2010). Linking empowering leadership and employee creativity: the influence of psychological empowerment, intrinsic motivation, and creative process engagement. *Academy of Management Journal*.

[8] Lee A., Willis S., Tian A. W. (2018). Empowering leadership: a meta-analytic examination of incremental contribution, mediation, and moderation. *Journal of Organizational Behavior*.

[9] Kim M., Beehr T. A., Prewett M. S. (2018). Employee responses to empowering leadership: a meta-analysis. *Journal of Leadership and Organizational Studies*.

[10] Kim D., Moon C. W., Shin J. (2018). Linkages between empowering leadership and subjective well-being and work performance via perceived organizational and co-worker support. *The Leadership and Organization Development Journal*.

[11] Tripathi N., Bharadwaja M. (2020). Empowering leadership and psychological health: the mediating role of psychological empowerment. *Employee Responsibilities and Rights Journal*.

[12] Cheong M., Spain S. M., Yammarino F. J., Yun S. (2016). Two faces of empowering leadership: enabling and burdening. *The Leadership Quarterly*.

[13] Kubicek B., Paškvan M., Bunner J., Korunka C., Kubicek B. (2017). The bright and dark sides of job autonomy. *Job Demands in a Changing World of Work: Impact on Workers’ Health and Performance and Implications for Research and Practice*.

[14] O’Connor D. B., Thayer J. F., Vedhara K. (2021). Stress and health: a review of psychobiological processes. *Annual Review of Psychology*.

[15] Lightman S. L., Birnie M. T., Conway-Campbell B. L. (2020). Dynamics of ACTH and cortisol secretion and implications for disease. *Endocrine Reviews*.

[16] Adam E. K., Quinn M. E., Tavernier R., McQuillan M. T., Dahlke K. A., Gilbert K. E. (2017). Diurnal cortisol slopes and mental and physical health outcomes: a systematic review and meta-analysis. *Psychoneuroendocrinology*.

[17] Hansen A. M., Garde A. H., Persson R. (2008). Sources of biological and methodological variation in salivary cortisol and their impact on measurement among healthy adults: a review. *Scandinavian Journal of Clinical and Laboratory Investigation*.

[18] El-Farhan N., Rees D. A., Evans C. (2017). Measuring cortisol in serum, urine and saliva – are our assays good enough?. *Annals of Clinical Biochemistry: International Journal of Laboratory Medicine*.

[19] Dickerson S. S., Kemeny M. E. (2004). Acute stressors and cortisol responses: a theoretical integration and synthesis of laboratory research. *Psychological Bulletin*.

[20] Kim M., Beehr T. A. (2018). Can empowering leaders affect subordinates’ well-being and careers because they encourage subordinates’ job crafting behaviors?. *Journal of Leadership & Organizational Studies*.

[21] Biggs A., Brough P., Drummond S., Cooper C. L., Quick J. C. (2017). Lazarus and folkman’s psychological stress and coping theory. *The Handbook of Stress and Health: A Guide to Research and Practice*.

[22] Daniels K., Beesley N., Cheyne A., Wimalasiri V. (2008). Coping processes linking the demands-control-support model, affect and risky decisions at work. *Human Relations*.

[23] Morin A., Arens A. K., Marsh H. W. (2016). A bifactor exploratory structural equation modeling framework for the identification of distinct sources of construct-relevant psychometric multidimensionality. *Structural Equation Modeling: A Multidisciplinary Journal*.

[24] Nicolson N. A., Luecken L. J., Gallo L. C. (2008). Measurement of cortisol. *Handbook of Physiological Research Methods in Health Psychology*.

[25] Weitzman E. D., Fukushima D., Nogeire C., Roffwarg H., Gallagher T. F., Hellman L. (1971). Twenty-four hour pattern of the episodic secretion of cortisol in normal subjects. *Journal of Clinical Endocrinology and Metabolism*.

[26] Barnett R. (2016). Cushing’s syndrome. *The Lancet*.

[27] Cougot B., Gauvin J., Gillet N. (2019). Impact at two years of an intervention on empowerment among medical care teams: study protocol of a randomised controlled trial in a large French university hospital. *Biomedical Central Health Services Research*.

[28] Wolf E. J., Harrington K. M., Clark S. L., Miller M. W. (2013). Sample size requirements for structural equation models: an evaluation of power, bias, and solution propriety. *Educational and Psychological Measurement*.

[29] Brossaud J., Leban M., Corcuff J.-B. (2018). LC-MSMS assays of urinary cortisol, a comparison between four in-house assays. *Clinical Chemistry and Laboratory Medicine*.

[30] Konczak L. J., Stelly D. J., Trusty M. L. (2000). Defining and measuring empowering leader behaviors: development of an upward feedback instrument. *Educational and Psychological Measurement*.

[31] Cougot B., Gillet N., Gauvin J. (2022). Impact of empowering leadership on emotional exhaustion: a controlled interventional study in a large French university hospital complex. *Journal of Nursing Management*.

[32] Cohen S., Kamarck T., Mermelstein R. (1983). A global measure of perceived stress. *Journal of Health and Social Behavior*.

[33] Vallejo M. A., Vallejo-Slocker L., Fernández-Abascal E. G., Mañanes G. (2018). Determining factors for stress perception assessed with the perceived stress scale (PSS-4) in Spanish and other European samples. *Frontiers in Psychology*.

[34] Marsh H. W., Muthén B., Asparouhov T. (2009). Exploratory structural equation modeling, integrating cfa and efa: application to students’ evaluations of university teaching. *Structural Equation Modeling: A Multidisciplinary Journal*.

[35] Buuren S. (2018). *Flexible Imputation of Missing Data*.

[36] Hayes A. F. (2009). Beyond Baron and Kenny: statistical mediation analysis in the new millennium. *Communication Monographs*.

[37] Zhang Z., Wang L., Tong X., van der Ark L. A., Bolt D. M., Wang W.-C., Douglas J. A., Chow S.-M. (2015). Mediation analysis with missing data through multiple imputation and bootstrap. *Quantitative Psychology Research*.

[38] Ming S., Zhang H., Zhang Z., Wang L. (2022). Bmemlavaan: an r package for estimating and testing mediation effect in mediation models. https://mirror.lyrahosting.com/CRAN/web/packages/bmemLavaan/vignettes/R_package-bmemLavaan_vignette.pdf.

[39] Lesage F.-X., Berjot S., Deschamps F. (2012). Psychometric properties of the French versions of the perceived stress scale. *International Journal of Occupational Medicine and Environmental Health*.

[40] Bakker A. B., Demerouti E. (2017). Job demands-resources theory: taking stock and looking forward. *Journal of Occupational Health Psychology*.

[41] Bawden D., Robinson L. (2009). The dark side of information: overload, anxiety and other paradoxes and pathologies. *Journal of Information Science*.

[42] Chénard Poirier L.-A., Morin A. J. S., Boudrias J.-S. (2017). On the merits of coherent leadership empowerment behaviors: a mixture regression approach. *Journal of Vocational Behavior*.

[43] Dewe P., Cooper C., Quick J. C. (2017). Demand, resources, and their relationship with coping: developments, issues, and future directions. *The Handbook of Stress and Health: A Guide to Research and Practice*.

[44] Hobfoll S. E. (1989). Conservation of resources: a new attempt at conceptualizing stress. *American Psychologist*.

[45] Aldao A., Nolen-Hoeksema S., Schweizer S. (2010). Emotion-regulation strategies across psychopathology: a meta-analytic review. *Clinical Psychology Review*.

[46] Ford M. B., Collins N. L. (2010). Self-esteem moderates neuroendocrine and psychological responses to interpersonal rejection. *Journal of Personality and Social Psychology*.

[47] Bowling N. A., Eschleman K. J., Wang Q., Kirkendall C., Alarcon G. (2010). A meta-analysis of the predictors and consequences of organization-based self-esteem. *Journal of Occupational and Organizational Psychology*.

