# Supplementary material for: Relationship between Empowering Leadership and Stress in a French University Hospital: A Cross-Sectional Study Combining the Measurement of Perceived Stress and Salivary Cortisol
Source: J Nurs Manag. 2024 Jan 31;2024:8839893. doi: 10.1155/2024/8839893 (PMC11919181; doi:10.1155/2024/8839893)
Supplement: Supplementary Materials — Supplementary Figure S1: alternative models for measuring empowering leadership. Supplementary Figure S2: distributions of the imputed (red) and observed (blue) cortisol levels. Supplementary Table S1: Leader Empowering Behavior Questionnaire (LEBQ). Supplementary Table S2: sample characteristics. Supplementary Table S3: standardized parameter estimates (loadings λ; residuals δ) for the CFA, BCFA, ESEM, and BESEM models for measuring empowering leadership. Supplementary Table S4: correlations between the latent factors in the CFA and ESEM models. Supplementary Table S5: the hierarchical linear model of perceived stress: distinction between the global factor and the specific factors for empowering leadership. Supplementary Table S6: bootstrapping analyses combined with multiple imputations for the test of the mediating effect of perceived stress. [file 8839893.f1.doc]

**Supplementary material**

**Online Supplements for:**

**Relationship between empowering leadership and stress: A cross-sectional study combining the measurement of perceived stress and salivary cortisol**

**Authors’ note**

These online technical appendices are to be posted on the journal website and hot-linked to the manuscript. If the journal does not offer this possibility, these materials can alternatively be posted on one of our personal websites (we will adjust the in-text reference upon acceptance).

We would also be happy to have some of these materials brought back into the main manuscript, or included as published appendices if you deem it useful. We developed these materials to provide additional technical information and to keep the main manuscript from becoming needlessly long.

Supplementary figure S1. Alternative models for measuring empowering leadership.

a. Confirmatory Factor Analysis (CFA)

b. Exploratory Structural Equation Modeling (ESEM)

c. Bifactor CFA (BCFA)

d. Bifactor ESEM (BESEM)

*Note:* F1=delegation of authority; F2=accountability; F3=self-directed decision making; F4=information sharing; F5=skills development; F6=coaching for innovative performance; FG=global factor; i=item.

Supplementary figure S2. Distributions of the imputed (red) and observed (blue) cortisol levels


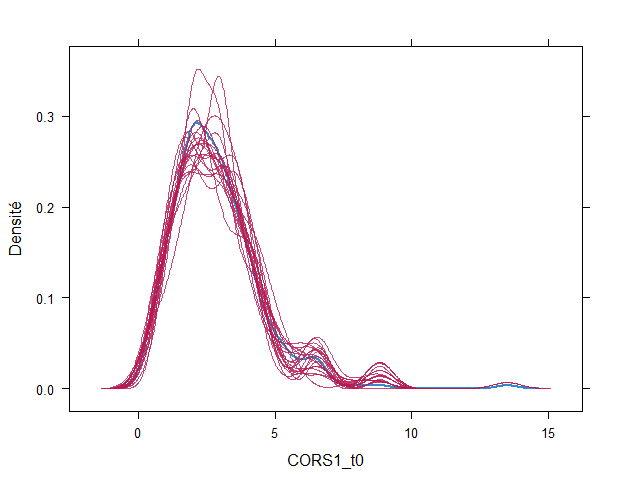


Density

Cortisol level (ng/mL)

| **Supplementary table S1. Leader Empowering Behavior Questionnaire (LEBQ)** | |
| --- | --- |
| *Dimensions* | *Items* |
| Delegation of authority | 1. My manager gives me the authority I need to make decisions that improve work processes and procedures. |
|  | 2. My manager gives me the authority to make changes necessary to improve things. |
|  | 3. My manager delegates to me the authority corresponding to the level of responsibility of my position. |
| Accountability | 4. My manager considers me responsible for the work I have to do. |
|  | 5. I am responsible for performance and results. |
|  | 6. My manager considers that the department staff is responsible for patient satisfaction. (contextualized) |
| Self-directed decision making | 7. My manager tries to help me find my own solutions in case of problems, rather than telling me what he/she would do |
|  | 8. My manager lets me make my own decisions about issues related to how the work is done. |
|  | 9. My manager encourages me to develop my own solutions to problems I encounter in my work. |
| Information sharing | 10. My manager shares the information I need to ensure high quality results. |
|  | 11. My manager provides me with the information I need to meet patients’ needs. (contextualized) |
| Skills development | 12. My manager encourages me to use systematic problem-solving methods (e.g., the seven-step problem-solving model). |
|  | 13. My manager frequently gives me the opportunity to develop new skills. |
|  | 14. My manager ensures that continuous learning and skills development are priorities in our department. |
| Coaching for innovative performance | 15. My manager is willing to let me take the risk of making mistakes if I learn from them and if they allow me to develop skills afterwards. |
|  | 16. I am encouraged to try out new ideas even if there is a possibility they do not work. |
|  | 17. My manager focuses on corrective action rather than blame when I make a mistake. |

| **Supplementary table S2. Sample characteristics** | | |
| --- | --- | --- |
| Corticosteroid therapy | |  |
|  | NA (%) | 3.27 |
|  | Yes (%) | 3.53 |
|  | No (%) | 93.20 |
| Gender | |  |
|  | Male (%) | 19.40 |
|  | Female (%) | 80.60 |
| Position | |  |
|  | Nurses (%) | 36.27 |
|  | Nursing assistants (%) | 22.17 |
|  | Physicians (%) | 21.91 |
|  | Secretaries (%) | 6.55 |
|  | Hospital service workers (%) | 3.27 |
|  | Nurse managers (%) | 4.79 |
|  | Head physicians (%) | 1.26 |
|  | Others (%) | 3.78 |
| Working time | |  |
|  | Full-time (%) | 71.54 |
|  | Part-time (%) | 28.46 |
| SC (mean ± SD) | | 2.84 ± 1.60 |
| PS (mean ± SD) | | 6.16 ± 3.25 |
| GF for EL (mean ± SD) | | 4.61 ± 0.95 |
| Delegation of authority (mean ± SD) | | 4.45 ± 1.46 |
| Accountability (mean ± SD) | | 5.50 ± 0.96 |
| Self-directed decision making (mean ± SD) | | 4.74 ± 1.23 |
| Information sharing (mean ± SD) | | 4.65 ± 1.45 |
| Skills development (mean ± SD) | | 4.17 ± 1.30 |
| Coaching for innovative performance (mean ± SD) | | 4.05 ± 1.32 |
| *Note:* %=percentage; SD=standard deviation; SC=salivary cortisol; PS=perceived stress; GF for EL=global factor for empowering leadership. The means and SD were calculated without imputing the missing data for SC, and directly from the item responses for the PS and EL dimensions. The PS scores ranged from 0 to 16. They were calculated by summing the items as described by Lesage et al. (2012). The scores for the EL dimensions ranged from 1 to 7. They were calculated by averaging the responses to the items as described by Konczak et al. (2000). | | |
|
|
|
|

| **Supplementary table S3. Standardized parameter estimates (loadings λ; residuals δ) for the CFA, BCFA, ESEM and BESEM models for measuring empowering leadership** | | | | | | | | | | | | | | | | | | | | |
| --- | --- | --- | --- | --- | --- | --- | --- | --- | --- | --- | --- | --- | --- | --- | --- | --- | --- | --- | --- | --- |
| *Items* | CFA |  | BCFA | |  | ESEM |  |  |  |  |  |  | BESEM |  |  |  |  |  |  |  |
|  | *λ* | *δ* | *GF-λ* | *SF-λ* | *δ* | *λ* | *λ* | *λ* | *λ* | *λ* | *λ* | *δ* | *GF-λ* | *SF-λ* | *SF-λ* | *SF-λ* | *SF-λ* | *SF-λ* | *SF-λ* | *δ* |
| Delegation of authority | | |  |  |  |  |  |  |  |  |  |  |  |  |  |  |  |  |  |  |
| Item 1 | 0.895 | 0.199 | 0.715 | 0.523 | 0.215 | **0.845** | -0.052 | *0.050* | *0.022* | *0.030* | *0.023* | 0.210 | **0.706** | **0.476** | *0.011* | *0.018* | *-0.008* | *0.011* | *0.046* | 0.272 |
| Item 2 | 0.914 | 0.165 | 0.708 | 0.623 | 0.111 | **0.949** | *-0.035* | *0.033* | *0.057* | *-0.009* | *-0.065* | 0.116 | **0.702** | **0.708** | *0.013* | *0.026* | *0.009* | *-0.016* | *-0.033* | 0.004 |
| Item 3 | 0.691 | 0.523 | 0.692 | 0.202 | 0.480 | **0.337** | 0.322 | *-0.013* | *0.090* | *-0.008* | 0.287 | 0.410 | **0.671** | **0.214** | 0.247 | *-0.031* | *0.028* | *-0.060* | *0.152* | 0.414 |
| *ω* | 0.876 |  |  | 0.693 |  | 0.861 |  |  |  |  |  |  |  | 0.739 |  |  |  |  |  |  |
| Accountability | | |  |  |  |  |  |  |  |  |  |  |  |  |  |  |  |  |  |  |
| Item 4 | 0.901 | 0.188 | 0.538 | 0.590 | 0.363 | 0.133 | **0.677** | *0.032* | *0.094* | *0.018* | *0.021* | 0.342 | **0.486** | *0.129* | **0.627** | *0.118* | 0.103 | *-0.027* | *0.116* | 0.315 |
| Item 5 | 0.641 | 0.590 | 0.337 | 0.665 | 0.445 | *0.009* | **0.748** | *-0.010* | *-0.083* | *0.053* | *0.042* | 0.449 | **0.376** | *-0.005* | **0.682** | *-0.005* | *-0.139* | *-0.048* | -0.172 | 0.342 |
| Item 6 | 0.360 | 0.870 | *0.057* | 0.485 | 0.761 | *-0.136* | **0.445** | *0.177* | *0.038* | *0.014* | -0.218 | 0.727 | ***0.053*** | *-0.024* | **0.427** | 0.250 | *0.063* | *-0.004* | *-0.111* | 0.736 |
| *ω* | 0.687 |  |  | 0.659 |  |  | 0.697 |  |  |  |  |  |  |  | 0.684 |  |  |  |  |  |
| Self-directed decision making | | | | | | |  |  |  |  |  |  |  |  |  |  |  |  |  |  |
| Item 7 | 0.654 | 0.572 | 0.649 | *0.161* | 0.553 | *0.097* | *0.030* | **0.300** | *0.132* | *-0.038* | 0.356 | 0.536 | **0.735** | *-0.084* | *-0.050* | ***0.073*** | *-0.116* | -0.151 | *-0.128* | 0.392 |
| Item 8 | 0.612 | 0.625 | 0.373 | 0.589 | 0.513 | 0.165 | 0.189 | **0.633** | *-0.088* | *-0.052* | -0.158 | 0.467 | **0.357** | 0.133 | 0.273 | **0.592** | -0.020 | *-0.060* | *-0.008* | 0.425 |
| Item 9 | 0.809 | 0.345 | 0.609 | 0.590 | 0.281 | *-0.069* | *-0.041* | **0.871** | *0.042* | 0.043 | *0.091* | 0.195 | **0.622** | -0.089 | *0.033* | **0.595** | *0.000* | *-0.001* | *0.010* | *0.250* |
| *ω* | 0.736 |  |  | 0.571 |  |  |  | 0.731 |  |  |  |  |  |  |  | 0.598 |  |  |  |  |
| Information sharing | | |  |  |  |  |  |  |  |  |  |  |  |  |  |  |  |  |  |  |
| Item 10 | 0.902 | 0.187 | 0.757 | 0.329 | 0.319 | *0.125* | *0.029* | *0.069* | **0.626** | *0.124* | *0.012* | 0.299 | **0.720** | *0.059* | *0.019* | *0.021* | **0.408** | 0.106 | *-0.012* | 0.300 |
| Item 11 | 0.824 | 0.321 | 0.695 | 0.669 | 0.069 | *0.028* | *-0.004* | *-0.030* | **0.911** | *0.032* | *-0.030* | 0.160 | **0.687** | *-0.026* | *-0.019* | *-0.049* | **0.598** | *0.067* | *-0.060* | *0.159* |
| *ω* | 0.854 |  |  | 0.720 |  |  |  |  | 0.837 |  |  |  |  |  |  |  | 0.688 |  |  |  |
| Skills development | | | |  |  |  |  |  |  |  |  |  |  |  |  |  |  |  |  |  |
| Item 12 | 0.526 | 0.724 | 0.508 | 0.169 | 0.714 | *-0.107* | *0.003* | 0.244 | 0.184 | **0.289** | *0.076* | 0.674 | **0.504** | -0.107 | *-0.034* | 0.142 | 0.096 | ***0.160*** | *-0.050* | 0.676 |
| Item 13 | 0.852 | 0.275 | 0.665 | 0.530 | *0.276* | *0.032* | *-0.001* | *0.008* | *0.006* | **0.852** | *-0.005* | *0.239* | **0.652** | *-0.007* | *-0.056* | *-0.030* | *0.034* | **0.646** | *-0.002* | *0.152* |
| Item 14 | 0.786 | 0.382 | 0.604 | 0.525 | 0.360 | *0.009* | *0.068* | -0.129 | *0.044* | **0.753** | *0.071* | 0.369 | **0.574** | *0.005* | *-0.020* | -0.109 | *0.099* | **0.467** | 0.124 | 0.416 |
| *ω* | 0.772 |  |  | 0.526 |  |  |  |  |  | 0.737 |  |  |  |  |  |  |  | 0.566 |  |  |
| Coaching for innovative performance | | | | | |  |  |  |  |  |  |  |  |  |  |  |  |  |  |  |
| Item 15 | 0.674 | 0.546 | 0.565 | 0.488 | 0.443 | 0.166 | *-0.077* | *0.123* | -0.149 | *0.185* | **0.493** | 0.529 | **0.577** | *0.070* | -0.140 | *0.011* | *-0.126* | *0.063* | **0.386** | 0.474 |
| Item 16 | 0.783 | 0.387 | 0.713 | 0.284 | 0.411 | *0.105* | *-0.004* | 0.146 | *0.064* | *0.248* | **0.369** | 0.456 | **0.682** | *0.044* | *-0.060* | *0.044* | *0.031* | 0.131 | **0.273** | 0.435 |
| Item 17 | 0.726 | 0.473 | 0.693 | 0.244 | 0.460 | *0.035* | *0.019* | *0.037* | 0.170 | *-0.012* | **0.671** | 0.351 | **0.728** | *-0.041* | -0.107 | -0.118 | *-0.004* | *-0.055* | **0.206** | 0.398 |
| *ω* | 0.772 |  | 0.935 | 0.440 |  |  |  |  |  |  | 0.638 |  | 0.940 |  |  |  |  |  | 0.364 |  |
| *Note:* CFA=confirmatory factor analysis; ESEM=exploratory structural equation modeling; BCFA=bifactor CFA; BESEM=bifactor ESEM; GF=global factor estimated as part of a bifactor model; SF=specific factor estimated as part of a bifactor model; λ=factor loading; δ=item uniqueness; ω=omega coefficient of model-based composite reliability. Target ESEM and B-ESEM factor loadings are indicated in bold; non-significant parameters (p >0.05) are marked in italic. | | | | | | | | | | | | | | | | | | | | |

| **Supplementary table S4. Correlations between the latent factors in the CFA and ESEM models** | | | | | | |
| --- | --- | --- | --- | --- | --- | --- |
| CFA |  |  |  |  |  |  |
|  | Dele. | Acc. | Self. | Info. | Skills. | Innov. |
| Delegation of authority | - |  |  |  |  |  |
| Accountability | 0.546 | - |  |  |  |  |
| Self-directed decision making | 0.635 | 0.576 | - |  |  |  |
| Information sharing | 0.661 | 0.480 | 0.626 | - |  |  |
| Skills development | 0.589 | 0.345 | 0.543 | 0.732 | - |  |
| Coaching for innovative performance | 0.739 | 0.403 | 0.691 | 0.723 | 0.768 | - |
|  |  |  |  |  |  |  |
| ESEM |  |  |  |  |  |  |
|  | Dele. | Acc. | Self. | Info. | Skills. | Innov. |
| Delegation of authority | - |  |  |  |  |  |
| Accountability | 0.406 | - |  |  |  |  |
| Self-directed decision making | 0.454 | 0.443 | - |  |  |  |
| Information sharing | 0.507 | 0.301 | 0.443 | - |  |  |
| Skills development | 0.499 | 0.189 | 0.406 | 0.622 | - |  |
| Coaching for innovative performance | 0.557 | 0.161 | 0.348 | 0.513 | 0.558 | - |
|  |  |  |  |  |  |  |
| Differences between the correlation coefficients (ESEM - CFA) | | | | | | |
|  | Dele. | Acc. | Self. | Info. | Skills. | Innov. |
| Delegation of authority | - |  |  |  |  |  |
| Accountability | -0.140 | - |  |  |  |  |
| Self-directed decision making | -0.181 | -0.133 | - |  |  |  |
| Information sharing | -0.154 | -0.179 | -0.183 | - |  |  |
| Skills development | -0.090 | -0.156 | -0.137 | -0.110 | - |  |
| Coaching for innovative performance | -0.182 | -0.242 | -0.343 | -0.210 | -0.210 | - |
|  |  |  |  |  |  |  |
| *Note:* CFA=confirmatory factor analysis; ESEM=exploratory structural equation modeling; Dele.=delegation of authority; Acc.=Accountability ; Self.=self-directed decision making; Info.=information sharing; Skills.=skills development; Innov.=coaching for innovative performance. All correlations are statistically significant (p <0.01). | | | | | | |

| **Supplementary table S5. Hierarchical linear model of perceived stress: Distinction between the global factor and the specific factors for empowering leadership** | | | | | | | | | | | | | | | | |
| --- | --- | --- | --- | --- | --- | --- | --- | --- | --- | --- | --- | --- | --- | --- | --- | --- |
|  |  | Mps0 | | |  | MpsFG | | |  | MpsFS | | |  | Mps1 | | |
|  |  | *b* | *s.e.* | *p* |  | *b* | *s.e.* | *p* |  | *b* | *s.e.* | *p* |  | *b* | *s.e.* | *p* |
| Intercept | | -0.006 | 0.275 | 0.983 |  | 0.186 | 0.270 | 0.492 |  | -0.076 | 0.271 | 0.778 |  | 0.142 | 0.263 | 0.589 |
|  |  |  |  |  |  |  |  |  |  |  |  |  |  |  |  |  |
| Male versus female | |  |  |  |  |  |  |  |  |  |  |  |  |  |  |  |
|  | Female | 0.019 | 0.141 | 0.894 |  | -0.012 | 0.137 | 0.931 |  | 0.065 | 0.138 | 0.639 |  | 0.031 | 0.132 | 0.815 |
| Age | | 0.009 | 0.007 | 0.186 |  | 0.004 | 0.007 | 0.557 |  | 0.010 | 0.007 | 0.154 |  | 0.004 | 0.007 | 0.527 |
| Position N-assistants versus | |  |  |  |  |  |  |  |  |  |  |  |  |  |  |  |
|  | versus HSW | -0.195 | 0.274 | 0.478 |  | -0.175 | 0.266 | 0.510 |  | -0.131 | 0.269 | 0.627 |  | -0.122 | 0.259 | 0.639 |
|  | Others | -0.454 | 0.260 | 0.082 |  | -0.482 | 0.253 | 0.057 |  | -0.445 | 0.259 | 0.087 |  | -0.491 | 0.249 | 0.050 |
|  | Nurse managers | -0.467 | 0.238 | 0.050 |  | -0.381 | 0.232 | 0.101 |  | -0.466 | 0.235 | 0.048 |  | -0.389 | 0.226 | 0.086 |
|  | Head physicians | -0.289 | 0.434 | 0.505 |  | -0.141 | 0.423 | 0.739 |  | -0.256 | 0.426 | 0.548 |  | -0.112 | 0.410 | 0.784 |
|  | Nurses | -0.166 | 0.125 | 0.184 |  | -0.182 | 0.121 | 0.135 |  | -0.176 | 0.125 | 0.159 |  | -0.206 | 0.120 | 0.087 |
|  | Physicians | -0.096 | 0.155 | 0.538 |  | -0.142 | 0.151 | 0.348 |  | -0.114 | 0.156 | 0.464 |  | -0.188 | 0.150 | 0.212 |
|  | Secretaries | 0.031 | 0.206 | 0.879 |  | -0.048 | 0.201 | 0.811 |  | 0.106 | 0.205 | 0.604 |  | 0.012 | 0.198 | 0.953 |
| Working full-time versus | |  |  |  |  |  |  |  |  |  |  |  |  |  |  |  |
|  | part-time | -0.192 | 0.109 | 0.079 |  | -0.189 | 0.106 | 0.075 |  | -0.164 | 0.107 | 0.125 |  | -0.159 | 0.103 | 0.123 |
| Seniority | | -0.014 | 0.007 | 0.052 |  | -0.010 | 0.007 | 0.156 |  | -0.014 | 0.007 | 0.053 |  | -0.010 | 0.007 | 0.165 |
|  |  |  |  |  |  |  |  |  |  |  |  |  |  |  |  |  |
| GF for EL | |  |  |  |  | **-0.234** | **0.048** | **0.000** |  |  |  |  |  | **-0.267** | **0.047** | **0.000** |
| SF for dele. | |  |  |  |  |  |  |  |  | -0.063 | 0.053 | 0.238 |  | -0.027 | 0.051 | 0.595 |
| SF for acc. | |  |  |  |  |  |  |  |  | -0.015 | 0.055 | 0.784 |  | 0.000 | 0.052 | 0.996 |
| SF for self. | |  |  |  |  |  |  |  |  | **0.143** | **0.056** | **0.011** |  | **0.168** | **0.054** | **0.002** |
| SF for info. | |  |  |  |  |  |  |  |  | 0.102 | 0.057 | 0.073 |  | **0.132** | **0.055** | **0.017** |
| SF for skills. | |  |  |  |  |  |  |  |  | 0.018 | 0.053 | 0.727 |  | 0.041 | 0.051 | 0.424 |
| SF for innov. | |  |  |  |  |  |  |  |  | **0.273** | **0.069** | **0.000** |  | **0.307** | **0.066** | **0.000** |
|  |  |  |  |  |  |  |  |  |  |  |  |  |  |  |  |  |
| R² | |  |  | 0.039 |  |  |  | 0.096 |  |  |  | 0.100 |  |  |  | 0.172 |
| ΔR² with Mps0 | |  |  | - |  |  |  | **0.057** |  |  |  | **0.062** |  |  |  | **0.133** |
| ΔR² with MpsFG | |  |  | - |  |  |  | - |  |  |  | 0.005 |  |  |  | **0.076** |
| ΔR² with MpsFS | |  |  | - |  |  |  | - |  |  |  | - |  |  |  | **0.071** |
|  | |  |  |  |  |  |  |  |  |  |  |  |  |  |  |  |
| *Note:* Significant parameters are indicated in bold (p ≤0.05); b=non-standardized regression coefficient; s.e.=standard error of the coefficient; p=p value; C-therapy=Corticosteroid therapy; N-assistant=nursing assistant; HSW=hospital service worker; SF=specific factor; GF=global factor; Dele.= Delegation of authority; Acc.=accountability; Self.=self-directed decision making; Info.=information sharing; Skills.=skills development; Innov.=coaching for innovative performance; EL=empowering leadership; PS=perceived stress; R²= percentage of variance explained; ΔR²= difference in variance explained. | | | | | | | | | | | | | | | | |

| **Supplementary table S6. Bootstrapping analyses combined with multiple imputation for the test of the mediating effect of perceived stress** | | | |
| --- | --- | --- | --- |
| Mediation | Estimate  for the mediating effect of PS | Confidence intervals | |
|  |  | lower | Upper |
| GF for EL 🡪 PS 🡪 cortisol | 0.003 | -0.044 | 0.052 |
| SF for dele. 🡪 PS 🡪 cortisol | -0.003 | -0.034 | 0.012 |
| SF for acc. 🡪 PS 🡪 cortisol | -0.002 | -0.023 | 0.033 |
| SF for self. 🡪 PS 🡪 cortisol | 0.002 | -0.026 | 0.032 |
| SF for info. 🡪 PS 🡪 cortisol | 0.000 | -0.011 | 0.024 |
| SF for skills. 🡪 PS 🡪 cortisol | 0.000 | -0.010 | 0.013 |
| SF for innov. 🡪 PS 🡪 cortisol | -0.008 | -0.067 | 0.043 |
| *Note:* The results are based on 1000 bootstrap samples and 20 imputations; level of confidence intervals is 95%; SF=specific factor; GF=global factor; Dele.= Delegation of authority; Acc.=accountability; Self.=self-directed decision making; Info.=information sharing; Skills.=skills development; Innov.=coaching for innovative performance; EL=empowering leadership; PS=perceived stress. | | | |
